# Periodontal status and provision of periodontal services in Malaysia: are we meeting population needs?

**DOI:** 10.1186/1472-6963-12-S1-P8

**Published:** 2012-11-21

**Authors:** Tuti N Mohd-Dom, Syed M Aljunid, Rizal A Manaf, Khairiyah A Muttalib, Ahmad SM Asari

**Affiliations:** 1 United Nations University International Institute for Global Health, Universiti Kebangsaan Malaysia Medical Centre, Jalan Yaacob Latif, 56000 Bandar Tun Razak, Malaysia; 2 Department of Dental Public Health, Faculty of Dentistry, Universiti Kebangsaan Malaysia, Kuala Lumpur, Malaysia; 3 International Training Centre for Casemix and Coding, Universiti Kebangsaan Malaysia, Kuala Lumpur, Malaysia; 4 Department of Community Health, Universiti Kebangsaan Malaysia Medical Centre, Kuala Lumpur, Malaysia; 5 Oral Health Division, Ministry of Health, Putrajaya, Malaysia

## Background

The absence of published literature on periodontal treatment needs and services in developing countries has undermined the significance of periodontal disease burden and impacts on patients and healthcare systems. This study analyses periodontal status and population treatment needs of Malaysians as well as patterns of periodontal services provided at public dental clinics.

## Methods

A retrospective approach to analysis of secondary data was employed. Data for population treatment needs were extracted from three decennial national oral health surveys for adults (1990, 2000 and 2010). Annual reports from the dental subsystem of the government Health Information Management System (HIMS) provided information on oral health care delivery for years 2006-2010. These reports were a summary of aggregated data and did not allow for further analysis other than reporting absolute numbers and frequency distributions.

## Results

There was a slight decline of periodontal disease prevalence between 1990 (92.8%) to 2000 (90.2%) but a sharp rise was observed in the 2010 survey (96.7%). In terms of severity, there was increase in the proportion of subjects demonstrating periodontal destruction in the 2010 survey after showing improvements in the 2000 survey. Parallel to this observation, percentage of subjects not requiring periodontal treatment (TN0) increased from 1990 to 2000, only to drop in 2010. An increase in patients’ attendance was observed alongside a growing uptake of periodontal-related procedures (62.2% in 2006 to 73.6% in 2010) as compared to non-periodontics treatment. However, only about 10% of periodontics procedures were surgeries with the most common being flap surgery. Counselling was the most common non-surgical procedure observed. The highest number of non-periodontics dental procedures done was restorations while the least was fixed prosthodontics.

## Conclusion

The rising periodontal treatment needs in the population do not seem to have been met. In spite of the upward trend of clinic attendance, the mix and distribution of treatment provided did not reflect the increasing needs for complex periodontal treatment. There is a need for a more efficient delivery of public health promotion strategies to meet oral health needs of the population.

